# Rapid Prototyping of Organ-on-a-Chip Devices Using Maskless Photolithography

**DOI:** 10.3390/mi13010049

**Published:** 2021-12-29

**Authors:** Dhanesh G. Kasi, Mees N. S. de Graaf, Paul A. Motreuil-Ragot, Jean-Phillipe M. S. Frimat, Michel D. Ferrari, Pasqualina M. Sarro, Massimo Mastrangeli, Arn M. J. M. van den Maagdenberg, Christine L. Mummery, Valeria V. Orlova

**Affiliations:** 1 Department of Anatomy and Embryology, Leiden University Medical Center, 2333 ZA Leiden, The Netherlands; D.G.Kasi@lumc.nl (D.G.K.); M.N.S.de_Graaf@lumc.nl (M.N.S.d.G.); C.L.Mummery@lumc.nl (C.L.M.); 2 Department of Human Genetics, Leiden University Medical Center, 2333 ZA Leiden, The Netherlands; J.P.M.S.Frimat@lumc.nl (J.-P.M.S.F.); A.M.J.M.van_den_Maagdenberg@lumc.nl (A.M.J.M.v.d.M.); 3 Department of Neurology, Leiden University Medical Center, 2333 ZA Leiden, The Netherlands; M.D.Ferrari@lumc.nl; 4 Department of Microelectronics, Delft University of Technology, 2628 CD Delft, The Netherlands; P.A.Motreuil-Ragot@tudelft.nl (P.A.M.-R.); P.M.Sarro@tudelft.nl (P.M.S.); M.Mastrangeli@tudelft.nl (M.M.)

**Keywords:** SU-8, photoresist, polydimethylsiloxane (PDMS), maskless photolithography, grayscale photolithography, backside exposure, low-cost microfabrication, digital micromirror device (DMD), PRIMO, organ-on-a-chip (OoC)

## Abstract

Organ-on-a-chip (OoC) and microfluidic devices are conventionally produced using microfabrication procedures that require cleanrooms, silicon wafers, and photomasks. The prototyping stage often requires multiple iterations of design steps. A simplified prototyping process could therefore offer major advantages. Here, we describe a rapid and cleanroom-free microfabrication method using maskless photolithography. The approach utilizes a commercial digital micromirror device (DMD)-based setup using 375 nm UV light for backside exposure of an epoxy-based negative photoresist (SU-8) on glass coverslips. We show that microstructures of various geometries and dimensions, microgrooves, and microchannels of different heights can be fabricated. New SU-8 molds and soft lithography-based polydimethylsiloxane (PDMS) chips can thus be produced within hours. We further show that backside UV exposure and grayscale photolithography allow structures of different heights or structures with height gradients to be developed using a single-step fabrication process. Using this approach: (1) digital photomasks can be designed, projected, and quickly adjusted if needed; and (2) SU-8 molds can be fabricated without cleanroom availability, which in turn (3) reduces microfabrication time and costs and (4) expedites prototyping of new OoC devices.

## 1. Introduction

Organ-on-a-chip (OoC) is a technology that emerged from lab-on-a-chip and refers to biomimetic models built on a microfluidic chip. OoC models are engineered by integrating (human) cells and tissues within a microdevice that contains a single or multiple cell culture compartments or microfluidic channels, sensors, and/or valves [1,2,3]. Combined with human induced pluripotent stem cells (hiPSCs), OoC technology can be used to reveal disease mechanisms and to perform drug discovery, especially by employing patient-specific cells in combination with matched (genetically repaired) isogenic controls [4]. Organ-specific cells can then be derived from hiPSCs using standardized differentiation protocols and can be cultured in specialized chips to develop human disease models [5].

OoC devices are predominantly made of polydimethylsiloxane (PDMS) because it is a widely available polymer, easy to work with, and compatible with live cells. PDMS is optically transparent, biocompatible, enables rapid replication of molds using soft lithography, and, in addition, offers facile bonding to different substrates to generate chips with enclosed microfluidic channels and chambers [1,6,7,8]. However, molds to perform soft lithography cannot be produced easily without specialized facilities and equipment. For example, microfabrication of photoresist-based molds requires access to a cleanroom, silicon wafers, custom photomasks, and specialized equipment and training to perform photolithography. These requirements are complicated, expensive and time-consuming, and are often only available in technical universities [6,9,10], thus difficult to access for scientists from universities without these facilities. Being able to create OoC prototypes and microfluidic devices rapidly and make them suitable for specific research questions could benefit researchers from both technical and non-technical universities. It could also further streamline the adoption of the technology because of its simplicity, much as occurred with 3D (bio) printing [11,12]. While very promising for prototyping microfluidic and OoC devices, the adoption of 3D printing technology by biomedical researchers is currently hampered by concerns of cytotoxicity, optical clarity, limited resolution, and impeded curing of PDMS [12,13,14,15,16]. Efforts have been made to simplify microfabrication using cleanroom-free methods and/or low-cost, transparent substrates. For instance, coverslip glass has been used as a photoresist substrate and combined with low-cost UV lamps and photomasks in a cleanroom-free approach [17]. Polyethylene terephthalate (PET) has also been used as a low-cost, transparent substrate for photoresists; when combined with backside UV exposure, this resulted in an innovative approach for fabricating multi-level microstructures [18]. However, the major drawback is that photomasks are still necessary to perform UV exposure of the photoresist.

An alternative to photomasks is “maskless photolithography”, where the required patterns are projected directly onto the photoresist [19,20,21]. This form of photolithography can be performed by spatial modulation of UV light using a digital micromirror device (DMD) and was first demonstrated in 1999 [22]. Similar to other maskless techniques, DMD-based systems can be used to perform direct writing of microfeatures to the photoresist [23,24,25,26,27,28]. For example, DMD-based systems were used with various substrates to produce microfabricated systems that include microfluidic and cell culture devices and optical microlenses [25,29,30,31,32,33]. In addition, microstructures with complex geometries and submicrometer features have been generated using DMD-based systems [34,35,36]. However, at present, most DMD setups are custom-made and therefore cannot be easily adapted or replicated by other groups without deep knowledge of optics, mechanics, electronics, and computer programming [37]. Furthermore, conventional maskless photolithography is routinely performed using silicon wafers [19,20,38]. The increased availability of commercial, easy-to-use DMD-based systems (e.g., Heidelberg Instruments maskless aligners and Alvéole Lab PRIMO) means researchers can have easier access to novel integrated platforms for photolithography. Such platforms offer improved turnaround times because of the elimination of physical photomasks, user-friendly interfaces, and reduced prototyping times [36]. For example, Heidelberg maskless aligners have been used to produce biosensors and microfluidic devices [39,40,41,42]. DMD-based systems in general, therefore, offer tremendous potential to improve the prototyping stages of device microfabrication.

Here, we developed a rapid and cleanroom-free OoC microfabrication process flow that can be adopted by virtually any laboratory and requires a minimum of specialized equipment. For this, we used a commercially available DMD-based system (PRIMO, Alvéole Lab, Paris, France) [38,43,44]. The PRIMO system was used previously for various applications, such as photopatterning of proteins and cells, hydrogel structuration, and fabrication of electron microscopy grids [43,44,45,46,47]. We optimized existing protocols to make it compatible with glass as a substrate for a negative photoresist (SU-8) instead of using silicon wafers, as has been described recently [38]. Since we use transparent glass substrates, we opted for backside exposure, a feature not possible in other commercially available DMD-based systems. SU-8 spin-coated substrates of sufficient quality were prepared in bulk for maskless photolithography. Microfabricated molds were mounted in small Petri dishes as an easy-to-use format suitable for PDMS-based soft lithography. We show that hiPSC-derived vascular cells (endothelial cells (ECs) and smooth muscle cells (vSMCs)) and neurons can be cultured inside the chips, providing evidence that these PDMS chips are able to support live cells. Finally, we demonstrate that we are able to control the height of SU-8 microstructures by using backside UV exposure and grayscale photolithography. This could be beneficial for rapid prototyping of channels of different heights and/or small features such as cone-like structures to fabricate microwells for microtissues or microchannels with smoothened surfaces for fabrication of microfluidic blood vasculature replicas [48].

Using the process flow presented here, PDMS-based microfluidic chips can be easily designed and adjusted in a vector graphics editor, and new microfabricated SU-8 molds and PDMS chips can be made within hours. This significantly reduces normal prototyping time compared with conventional microfabrication. Because of the simplified microfabrication, fast adjustments to chip designs can be made. When required, finalized designs can be transferred to large-scale facilities for conventional cleanroom-based microfabrication.

## 2. Materials and Methods

### 2.1. Substrate Preparation

Glass 50 mm diameter coverslips (Menzel-Gläser #1, Thermo Fisher Scientific, Waltham, MA, USA) were cleaned using ultrasonic baths of acetone and isopropanol. Coverslips were submerged in a glass Petri dish filled with acetone. The Petri dish with coverslips was then sonicated in an ultrasonic bath for 10 min, after which the coverslips were quickly transferred to a second Petri dish with fresh acetone, and sonication was again performed for 20 min. Afterward, these steps were repeated with two sequential isopropanol baths. Coverslips were stored in isopropanol until usage.

Before coating substrates with SU-8 (Kayaku Advanced Materials, Inc., Berlin, Germany), coverslips were retrieved from the isopropanol and dried with a nitrogen gun, after which a dehydration bake was performed on a hotplate at 200 °C for 5 min.

### 2.2. SU-8 Spin Coating and Soft Bake

Depending on the required microstructure height, SU-8 2075 or SU-8 2005 was used. Both types of SU-8 were deposited on the substrates using lubricant-free 60 mL syringes (Henke-Ject, Henke-Sas Wolf, Tuttlingen, Germany) with a partially cut-off tip to improve the outflow rates.

All spin coating and baking steps were performed in a fume hood. Petri dishes (145 mm diameter) were used as a cover to protect the substrates from dust particles and light. Briefly, the inner surface of 145 mm Petri dishes was coated with a thin layer of polydimethylsiloxane (PDMS, Sylgard 184, 10:1 base–curing agent, Dow Corning, Midland, MI, USA) and subsequently cured at 70 °C for 3 h, after which the exterior of the Petri dishes was wrapped in aluminum foil. The PDMS layer allows efficient protection of the substrate from dust particles, and aluminum foil protects from light.

To improve the adhesion of SU-8 to the glass substrates, SU-8 2005 was used as an adhesion enhancer prior to coating with the desired type of SU-8. Briefly, 2 mL of SU-8 2005 were deposited in the center of the dehydrated glass coverslips. Spin coating (WS-650MZ-23NPP, Laurell Technologies, North Wales, PA, USA) was performed at 2000 rpm, followed by a soft bake for 2 min at 65 °C and 4 min at 95 °C. The substrates were then allowed to cool down to room temperature (RT) on the hotplate and were subsequently overexposed for 5 min using a UV lamp (ATI26D, Alpha Innotech Corp, San Leandro, CA, USA). A post-exposure bake was performed afterward for 2 min at 65 °C and 2 min at 95 °C. The substrates were then allowed to cool down to RT on the hotplate after the baking step was completed and stored in the dark until use.

For coating the SU-8 layer for fabrication of microstructures, precoated coverslips were rinsed with absolute ethanol and dried with a nitrogen gun. Immediately afterward, 2 mL of either SU-8 2075 or SU-8 2005 were deposited in the center. For SU-8 2075, the coverslips were tilted at a 45-degree angle and slowly rotated to spread the SU-8 over the coverslip, leaving 5 mm from the edge uncoated. This was done to wet the coverslip with viscous SU-8 2075 and to ensure that a homogeneous layer was present after the spin coating step. Coverslips were then placed on a level surface for 2 min to allow the SU-8 to reflow.

Spin coating and subsequent soft baking were performed as follows:

1. Spin coating
  (a) 500 rpm for a total of 10 s with 100 rpm/s acceleration
  (b) 1000, 2000, 3000, or 4000 rpm held for 30 s with 300 rpm/s acceleration
  (c) 300 rpm/s deceleration until stop
2. Soft bake

Soft bake was performed on a hotplate that was manually controlled to obtain the required temperatures. Baking times and temperatures are indicated below (Table 1 and Table 2) and were optimized to prevent bowing/deformation of the thin glass substrates due to thermal stress in the spin-coated SU-8 layer. It should be noted that the hotplate was allowed to cool to RT with the substrates on top after the baking step was completed, as this also aided in preventing thermal stress.

### 2.3. Maskless Photolithography

Substrates were placed into an adjustable microscope holder for backside UV exposure. To expose SU-8 coated substrates, a maskless DMD-based photolithography system (PRIMO, Alvéole Lab) connected to a Leica DMi8 inverted microscope was used.

A motorized microscope stage (SCAN^plus^ IM 130 × 85, Märzhäuser Wetzlar, Wetzlar, Germany), connected to a Tango controller (Märzhäuser Wetzlar) was controlled by Leonardo software (Alvéole Lab) for automated stitching of areas of exposure. Binary digital photomasks for chip and microfeatures were designed in an open-source vector graphics editor (Inkscape). Digital photomasks were then loaded into the Leonardo software (Alvéole Lab) and projected by the system using a DMD and 375 nm UV laser at 200 mW direct output power via either a 2.5X/0.07NA, 5X/0.15NA, or 20X/0.40NA objective, depending on the required dimensions of the microstructures. Various laser doses were used for SU-8 substrates, depending on the thickness of the SU-8 layer (Supplementary Table S1). Grayscale photolithography was performed by projection of 8-bit grayscale digital photomasks at laser doses of 2–6 mJ/mm^2^.

### 2.4. Post-Exposure Bake, Development, and Hard-Bake

After exposure, substrates were post-exposure baked on a hotplate that was manually controlled to obtain the required temperatures. Baking times and temperatures are indicated below (Table 3 and Table 4) and were optimized for the same reasons as described above for soft baking.

Substrates were subsequently developed for 10 min by immersion in propylene glycol methyl ether acetate (PGMEA, 484431, Sigma-Aldrich, St. Louis, MI, USA), with gentle agitation. For single and double SU-8 2075 layers generated using 1000 rpm, a development time of 40 min was used.

Finally, to improve the durability of the molds and to anneal cracks, the SU-8 molds were subjected to a hard-bake at 120 °C for 20 min. The substrates were allowed to cool down to RT on the hotplate afterward.

### 2.5. Realignment Procedure for Fabrication of the Multi-Level Neuron Chip

After spin coating the substrate with SU-8 2005 (4000 rpm, intended for fabricating the lower microchannels) and soft baking, the substrate was gently clamped between the adjustable parts of the microscope holder. The holder was then placed inside the microscope stage connected to the PRIMO system. Before exposure, markers were placed (using a fine tip black marker pen) in four corners, as shown in Supplementary Figure S1A(i) (blue arrows). These markers spanned both parts of the substrate and the microscope holder (Supplementary Figure S1A(ii), blue and red arrows). In this way, the coarse position of the substrate could be restored later (as described below).

Subsequently, the whole substrate was imaged using Leonardo software in order to store the exact position of the substrate (Supplementary Figure S1B(i)). Exposure with the microchannel patterns was then performed (5X objective), after which the holder was removed from the microscope stage. It should be noted that the Leonardo software stores the location of previously used exposure patterns. The substrate was then carefully removed from the holder without moving the adjustable parts (to retain their position).

Next, a post-exposure bake of the SU-8 2005 layer and subsequent spin coating of the SU-8 2075 layer (3000 rpm, intended for fabricating the higher main channels) on top of the SU-8 2005 layer were performed. After soft baking, the substrate was carefully returned to the microscope holder, again without moving the adjustable parts. The substrate was then carefully rotated to realign the markers (Supplementary Figure S1A(ii)) in order to restore the coarse initial position of the substrate. Finally, the holder was returned to the microscope stage.

Landmarks, as shown in Supplementary Figure S1B(ii) (blue arrows), were then located and used to restore the exact location of the substrate. Tweezers allowed careful movement of the substrate in the holder to perform realignment, guided by the live camera view (Supplementary Figure S1B(ii), non-shaded views) and previously imaged substrate view (Supplementary Figure S1B(ii), shaded views).

After realignment, exposure of the main channel patterns was performed (5X objective), adjacent to the area already exposed, containing the microchannel patterns. The substrate was then removed from the holder, and a post-exposure bake was performed. The SU-8 could then be developed in a single step to obtain the multi-level microstructures.

### 2.6. Scanning Electron Microscopy and Optical Profilometry

Samples were characterized by both scanning electron microscopy (SEM) and profilometry. SEM studies were performed using a Regulus 8230 Field-Emission Scanning Electron Microscope (Hitachi Hi-Tech Corp., Tokyo, Japan). Profilometry was performed by using a Dektak-8 stylus profilometer (Veeco, Plainview, NY, USA) and VK-X250 3D laser scanning confocal microscope (Keyence Corp., Osaka, Japan).

### 2.7. Soft Lithography and Chip Assembly

To fabricate PDMS chips using soft lithography, SU-8 molds were mounted in 60 mm diameter polystyrene Petri dishes (Greiner Bio-One, Frickenhausen, Germany). Briefly, PDMS was mixed in a 10:1 (base–curing agent) mass ratio, after which the bottom of the Petri dish was spin coated with 1 g of PDMS at 4000 rpm held for 30 s with 300 rpm/s acceleration. Subsequently, SU-8 molds with microstructures facing up were gently pressed against the bottom of the Petri dish, after which the Petri dish was filled with 10 g of PDMS. Petri dishes containing SU-8 molds and PDMS were then degassed for 1 h at RT under vacuum and cured at 70 °C for 3 h.

After curing, PDMS was allowed to cool down to RT and was carefully cut circumferentially at 5 mm from the sides of the Petri dish, after which PDMS was gently peeled off. A flowchart of the soft lithography procedure is presented in Supplementary Figure S2.

PDMS chips were cut to fit 50 mm diameter glass coverslips, and inlet and outlet holes were punched using a 2 mm biopsy puncher. Finally, the chips and coverslips were pretreated with air plasma (50 KHz, 50 W) for 1 min (CUTE, Femto Science Inc., Gyeonggi-do, Korea), after which the chips were contact bonded onto the glass coverslips.

### 2.8. Cell Culture

The following hiPSC lines were used: LUMC0054iCTRL (European human Pluripotent Stem Cell Registry (hPSCreg.eu) number LUMCi001-A) [49], FLB243 (hPSCreg number LUMCi028-A) [50] and LUMC0114iCTRL01 (hPSCreg number LUMCi003-A) [51]. hiPSC lines were maintained on Vitronectin-coated plates in TeSR-E8 medium (05940, StemCell Technologies, Vancouver, BC, Canada) and passaged once a week using Gentle Cell Dissociation Reagent (07174, StemCell Technologies). hiPSC-ECs and hiPSC-vSMCs were derived and maintained as previously described [52,53]. hiPSC-derived neurons were generated from neural progenitor cells (NPCs). hiPSC-NPCs were first derived and maintained using the STEMdiff SMADi Neural Induction Kit (05835, StemCell Technologies). hiPSC-derived neurons were then derived from NPCs and maintained using the STEMdiff Midbrain Neuron Differentiation Kit (100-0038, StemCell Technologies).

### 2.9. On-Chip Cell Culture

#### 2.9.1. Seeding and Culture of hiPSC-ECs in Microfluidic Chip

The microfluidic chips for the ECs were coated using a polydopamine (H8502, Sigma-Aldrich) and fibronectin (F1141, Sigma-Aldrich) coating. Immediately following plasma bonding of the chips to glass, the microfluidic channels were injected with 2 mg/mL dopamine in 10 mM Tris-HCL buffer (pH = 8.5) and incubated for 1 h at RT. Afterward, the channels were washed 3 times with PBS (without Ca^2+^ and Mg^2+^(-)). Then, 50 µg/mL of bovine plasma fibronectin in PBS (-) was added to the channels and incubated for 1 h at RT. Microfluidic channels were washed 3 times with PBS (-) afterward. ECs were harvested using TrypLE and resuspended in complete EC-SFM medium at a concentration of 2 × 10^4^ cells/µL. Then, 4 µL of cell suspension was added to the microfluidic channel that was prefilled with complete EC-SFM medium. Cells were allowed to attach for 2 h at 37 °C, and chips were then topped with full EC-SFM medium. After 24 h, chips were transferred to a rocking platform (OrganoFlow L, MIMETAS B.V., Leiden, The Netherlands) and cultured for 48 h under bidirectional flow (7-degree inclination at 8 min intervals).

#### 2.9.2. Seeding and Culture of hiPSC-vSMCs on Microgrooves

The PDMS (either with or without) microgrooves were coated using a polydopamine and fibronectin coating. After plasma treatment, the PDMS was covered with 2 mg/mL dopamine in 10 mM Tris-HCL buffer (pH = 8.5) and incubated for 1 h at RT. Afterward, the PDMS was washed 3 times with PBS (-). Then, 50 µg/mL of bovine plasma fibronectin in PBS (-) was added on top for 1 h at RT. PDMS was washed 3 times with PBS (-) afterward. vSMCs were harvested using TrypLE and resuspended in BPEL medium at a concentration of 3.5 × 10^3^ cells/µL, and 100 µL of cell suspension was then added on top of the PDMS. Cells were allowed to attach for 2 h at 37 °C, and PDMS was transferred to a well of a 6-well plate containing 2 mL of BPEL medium.

#### 2.9.3. Seeding and Culture of hiPSC-Neurons in Multi-Level Chips

Immediately following plasma bonding of the chips to glass, the microfluidic channels for the cortical neurons were injected with 100 µg/mL Poly-L-Ornithine (P3655, Sigma-Aldrich) and incubated at RT overnight. Prior to laminin coating (L2020, Sigma-Aldrich), chips were incubated at 4 °C for 30 min. Channels were washed with cold PBS (-), and laminin coating was then performed using a 200 µg/mL solution and subsequently incubated at 37 °C for 2 h. Chips were washed with PBS (-) and prefilled with a full STEMdiff midbrain neuron differentiation medium. Neurons were harvested using Accutase and were then resuspended at 10^4^ cells/µL in STEMdiff midbrain neuron differentiation medium. Then, 5 µL of cell suspension was injected in the channels and incubated for 2 h at 37 °C, and chips were topped with an additional STEMdiff midbrain neuron differentiation medium. After 3 days, the media were switched to full BrainPhys hPSC Neuron kit media (05795, StemCell Technologies) for the remainder of the experiment.

### 2.10. Immunofluorescence Staining

Cells were fixed by adding 4% paraformaldehyde (PFA) solution into the channels and incubated for 15 min at RT. For vSMCs on PDMS, a 100 µL droplet of 4% PFA was carefully added on top of the cells. Cells were then permeabilized for 10 min at RT using 0.1% Triton X-100 in PBS (-). Subsequently, cells were blocked for 1 h at RT using 1% bovine serum albumin (BSA) in PBS (-). Primary antibodies were diluted 1:200 in 1% BSA, injected into the channels, and incubated overnight at 4 °C. The primary antibodies used were against CD31 (PECAM1, mouse, M0823, Agilent Dako, Santa Clara, CA, USA) and MAP2 (mouse, ab11267, Abcam, Cambridge, UK) to stain ECs and neurons, respectively. For staining of F-actin in vSMCs, phalloidin was used (ActinGreen 488 ReadyProbes, R37110, Thermo Fisher Scientific). Samples were washed 3 times with PBS (-) afterward. Secondary antibody (A-21203, Thermo Fisher Scientific) was then diluted 1:300 in 1% BSA, injected into the channels and incubated at RT for 2 h. For the neurons in the chip with channels of different heights, an overnight incubation at 4 °C was performed. Cell nuclei were stained using Hoechst 33342 (NucBlue Live ReadyProbes, R37605, Thermo Fisher Scientific). Samples were stored in PBS (-) in the dark at 4 °C until imaging.

### 2.11. Fluorescence Imaging

Immunostained cells were imaged using a Leica SP8 microscope connected to a Dragonfly 500 spinning disk confocal system (Andor Technology Ltd., Belfast, Northern Ireland) with a 20X/0.75NA objective. Immunofluorescence overview scans were acquired using an EVOS M7000 microscope (Thermo Fisher Scientific) with a 10X/0.30NA objective.

## 3. Results and Discussion

### 3.1. Cleanroom-Free Microfabrication Process Flow for Organ-on-a-Chip Devices

**Figure 1:**
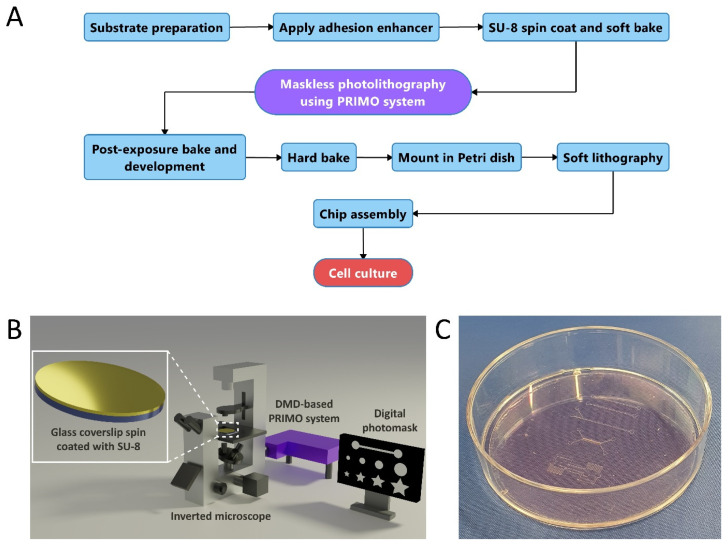
Microfabrication process flow using maskless photolithography. (**A**) Flowchart showing the developed approach to rapidly fabricate OoC devices. (**B**) Schematic of DMD-based maskless photolithography setup (PRIMO). (**C**) Photograph of microfabricated SU-8 mold mounted into a Petri dish (60 mm diameter) for soft lithography.

A schematic outline of the cleanroom-free microfabrication process flow for maskless photolithography is shown in Figure 1A. We used the commercially available PRIMO system (Alvéole Lab) to generate SU-8 microstructures reproducibly on glass substrates using 375 nm UV light (Figure 1B). Since the setup is based on a DMD connected to an inverted microscope, we opted for round glass coverslips of 130–160 μm thickness and 50 mm diameter as the substrate for SU-8. Compared to a silicon wafer, a glass coverslip has the advantage that it easily fits into adjustable microscope sample holders and enables proper focusing of the microscope optics through the thin glass onto the layer of SU-8. Backside UV exposure, therefore, becomes a possibility, eliminating the need for edge bead removal (EBR) and, subsequently, errors in the DMD projection of the digital photomask since the substrate is level. In order to improve the adhesion of SU-8 to the glass substrate, coverslips were first spin coated with SU-8 2005, followed by overexposure with UV. As a result, a thin adhesive layer of SU-8 was formed. After this step, an additional layer of SU-8 used for microfabrication was coated on top of the adhesive SU-8 layer. This approach is known to improve the durability of the mold and enables repeated PDMS replica molding [54]. After exposure of the digital photomask and subsequent processing, the glass substrate containing patterned microstructures is mounted into a 60 mm diameter polystyrene Petri dish (Figure 1C) which serves as a holder for the substrate and allows PDMS-based soft lithography.

Combining glass substrates with cleanroom-free photolithography as a low-cost alternative to conventional microfabrication has been demonstrated before using glass coverslips, inexpensive photomasks, and simple UV lamps [17]. It was shown that SU-8 microstructures for microfluidic applications can be patterned on glass using a cleanroom-free approach and simple equipment. However, laser-printed photomasks still need to be produced in order to expose SU-8. We have overcome this limitation by employing the maskless PRIMO system, where digital photomasks can be designed and projected by a DMD onto substrates directly [24,25,27,55] with a lateral resolution of 2 µm [43]. The process flow described here enables fabrication and cell seeding of functional OoCs within one working day (turnaround time of 6–8 h, Supplementary Table S2).

By using this approach: (1) digital photomasks can be designed and adapted easily if needed; (2) SU-8 molds can be fabricated without the need for a cleanroom, which (3) reduces microfabrication time and costs, and (4) expedites prototyping of new OoC devices.

### 3.2. Generation of SU-8 Microstructures Using Maskless Photolithography and Backside UV Exposure

**Figure 2:**
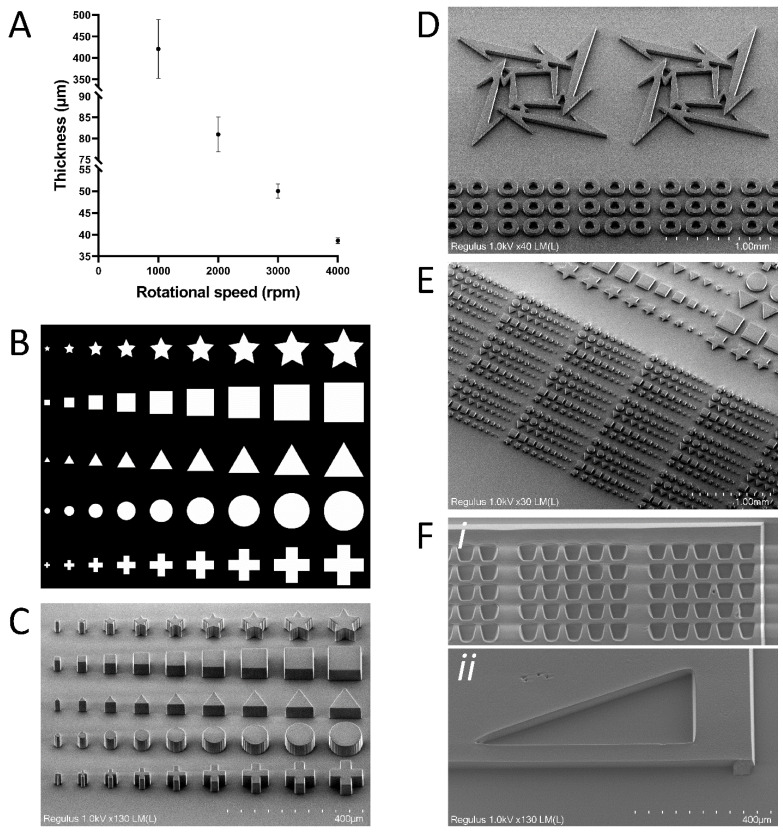
SU-8 microstructures generated using maskless photolithography on glass substrates. (**A**) Graph of obtained SU-8 layer thickness against the used spin coating speed. (**B**) Digital photomask design that was used for backside UV exposure using the PRIMO system and a 5X objective. (**C**) SEM images of 50 µm tall structures of various geometries that were microfabricated with high accuracy, as evident by the sharp edges and high reproducibility. (**D**) SEM image of more complex structures. These large structures were generated by the auto-stitching feature of the system, enabling the generation of large and continuous structures. (**E**) SEM image showing that whole arrays of microstructures can be microfabricated in large areas on the substrates. (**F**) SEM image of properly developed SU-8 that was exposed to inverted patterns. (i) SU-8 structures to generate pillars for hydrogel patterning. (ii) Acute angles are properly developed.

In order to generate structures of defined height, the thickness of spin coated SU-8 layers on the glass substrates was measured. Each type of SU-8 has a specific thickness range that is dependent on the rotational speed. In addition, the thickness range obtained after spin coating of SU-8 at specific rotational speeds can differ between labs due to external factors that may include environmental temperature, the method used for soft baking (e.g., type of hotplate or whether an oven is used), and the size of the substrate [17,56,57,58]. Therefore, the thickness range of SU-8 2075 was determined after spin coating at RT (19–22 °C) at various rotational speeds (1000 rpm, 2000 rpm, 3000 rpm, and 4000 rpm) (Figure 2A). A thickness range (from 39 μm to 420 μm) was obtained depending on the rotational speed (from 4000 rpm to 1000 rpm). At a lower rotational speed (1000 rpm), a larger variation in SU-8 layer thickness was obtained (420 μm ± 69 μm). Lower rotational speeds resulted in a SU-8 reflow effect and more prominent edge beads, causing SU-8 to reflow towards the center of the glass substrate during the soft bake step. The reflow effect combined with the low surface area of the substrate could potentially explain the higher SU-8 layer thickness variation obtained for low rotational speeds [56,58]. SU-8 2075 layer thickness of 50 μm (spin coated with a rotational speed of 3000 rpm) was therefore used to fabricate most of the structures unless indicated otherwise.

We first found that a laser dose of 6 mJ/mm^2^ was sufficient to expose SU-8 of 40–50 µm thickness. In addition, a 5X objective was used because it provides a proper balance between resolution and patterning speed since the field of view (FoV) is relatively large (2000 µm × 1250 µm), and one FoV takes only 4 s to expose. The Leonardo software (Alvéole Lab) that controls the PRIMO system enables imaging of the substrate, placement of digital photomasks, configuration of exposure dose, and auto-stitching of FoVs to enable exposure of large continuous patterns. On average, it takes less than 10 min to pattern substrate areas of 4 cm^2^.

Next, various geometries were tested to pattern SU-8 using backside UV exposure with the DMD-based maskless photolithography setup. The size of tested features ranged from 20 µm to 100 µm in width, with a fixed height of 50 µm, and these were projected using a digital photomask (Figure 2B). Scanning electron microscopy (SEM) revealed that microstructures as small as 20 µm could be obtained with straight walls and sharp edges (Figure 2C). In circular and non-rectangular features (e.g., stars, triangles, and circles), it was observed that the walls of these features were not smooth but striated. This effect is due to the rasterized projection of the patterns by the DMD [23] and was expected. More complex and larger structures with acute angles are also properly patterned (Figure 2D). These structures were generated using the auto-stitching feature of the PRIMO system, showing the feasibility of generating larger and continuous microstructures. Such large continuous structures are essential for the fabrication of OoC devices. Finally, again using the auto-stitching feature of the PRIMO system, we were able to generate an array of microstructures on large areas (2 cm × 2 cm) with high reproducibility (Figure 2E), and thereby demonstrate the feasibility of both (1) SU-8 layer preparation and (2) maskless photolithography on large glass substrate areas.

Inverted patterns of exposure are harder to fabricate because of the limited penetration of the SU-8 developer [56,59]. However, we have demonstrated that such structures (donut-like circles, Figure 2D) were properly developed. Additionally, we showed structures that can be used to generate on-chip PDMS pillars for hydrogel patterning (Figure 2F(i)) and that structures with sharp angles (Figure 2F(ii)) were properly developed. The digital photomask used in this study is shown in Supplementary Figure S3. It should be noted that particles cannot be eliminated completely because of the cleanroom-free approach. Even though we covered the substrates during every step of the process flow to minimize exposure to open air and light, particles could still fall on the substrates. For example, this can be observed in the top-left corner of Figure 2F(ii), where a small defect is caused by particles in the otherwise smooth SU-8 structure.

Furthermore, we were able to generate tall structures by using sequential SU-8 2075 layer coating at 1000 rpm rotational speed and exposure using a 2.5X objective. The 2.5X objective with low NA (0.07) has a relatively large depth-of-field and therefore enables the fabrication of tall structures with straight sidewalls as evident by 3:1 aspect ratio pillars of ~850 µm in height (Supplementary Figure S4A). Additionally, we fabricated structures that can be used to make microfluidic chips with high channels (Supplementary Figure S4B). Therefore, this approach could be an alternative to commonly used methods (e.g., micro-milling and 3D printing) for the fabrication of channels with a larger height for various applications, including organoid trapping [60]. Finally, to demonstrate the ability to fabricate microstructures in the range of the system’s lateral resolution limit (2 µm) using our process flow, we microfabricated ~6 µm-high “barcode-like” structures (SU-8 2005 spin coated at 4000 rpm) using a 20X/0.4NA objective (Supplementary Figure S5A). Supplementary Figure S5B shows an SEM overview image of the microstructures of increasing width (2–10 µm) that are 100 µm in length and ~6 µm high. This small array of structures was fabricated by using a single FoV. In Supplementary Figure S5C,D, it can be seen that the 2 µm wide structures are not straight when compared to the wider structures. This is due to the 3:1 aspect ratio of these small structures that increases their tendency to deform. This limitation can be overcome by optimizing the development time and minimizing agitation during development. When the line width increases (4–10 µm), the structures are stabilized and do not deform.

### 3.3. Fabrication of Microfluidic Channels and Microgrooves

To demonstrate that large SU-8 structures of sufficient quality for OoC applications can be microfabricated using this process flow, we next attempted to fabricate microfluidic chips (12 mm length, 300 µm width, and 50 µm height) and microgrooves (6 µm deep and 20 µm wide grooves, with 50 µm wide ridges).

**Figure 3:**
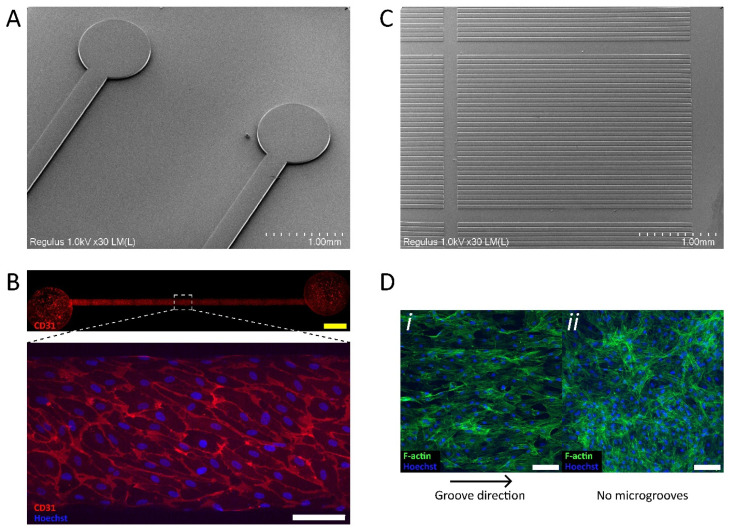
Fabrication of microchannels and microgrooves. (**A**) SEM image of 50 µm tall SU-8 structures to fabricate straight microfluidic channels 12 mm in length. (**B**) Representative immunofluorescence image of hiPSC-derived ECs upon 3 days of culture in microfluidic chip fabricated using SU-8 mold shown in A. ECs display typical morphology with an endothelial-specific CD31 marker located at the cell-cell junctions. (**C**) SEM image of 6 µm deep SU-8 microgrooves 5 mm in length (fabricated with a 20X objective). Grooves are 20 µm wide, while ridges are 50 µm wide. (**D**) Representative immunofluorescence image of hiPSC-derived vSMCs stained with phalloidin (F-actin) upon 5 days of culture on PDMS microgrooves fabricated using SU-8 mold shown in C (microgrooves are now inversed and are 50 µm wide, while ridges are 20 µm wide). (i) Aligned vSMCs cultured on microgrooves and (ii) non-aligned vSMCs cultured on flat PDMS that lacks microgrooves, as evident by F-actin staining. White scale bars: 100 µm. Yellow scale bar: 1000 µm.

Straight microfluidic channels were microfabricated using a 5X objective and the auto-stitching feature of the system (Figure 3A). After successful soft lithography and proper chip bonding to glass coverslips, hiPSC-derived ECs were seeded into the channels of fabricated chips and cultured for 24 h under static conditions and subsequently for 48 h under bidirectional flow on a rocking platform. Figure 3B shows that the microfluidic channel is completely covered with ECs and that the cells are still confluent in the chip even after 3 days of culture. In addition, the endothelial-specific CD31 marker is abundantly present at cell-cell junctions, indicating stable EC interaction. This shows that the process flow can be used to produce chips for OoC applications, despite the fact that particles might cause small defects in the SU-8 structures as described earlier (Figure 2F(ii)).

An array of microgrooves was generated by employing a 20X objective and the auto-stitching feature of the system (Figure 3C) using SU-8 2005 spin coated at 4000 rpm. The grooves are properly developed, and therefore, auto-stitching with a 20X objective can be used to fabricate an array of continuous microgrooves 5 mm in length and width ≤ 20 µm. After soft lithography, PDMS with inverse microgrooves (6 µm deep and 50 µm wide, with 20 µm wide ridges) was obtained. In order to induce alignment of vSMCs using topographical cues [61,62], hiPSC-vSMCs were cultured on microgrooves for 5 days (Figure 3D). Under these conditions, hiPSC-vSMCs showed alignment of actin fibers (visualized using F-actin) parallel to the groove direction (i) when compared to a flat PDMS culture surface without microgrooves (ii).

Our approach enables the facile fabrication of microfluidic chips and surfaces with topographical cues. Such features are of interest in both OoC and tissue engineering research [63,64,65]. In addition, the process flow is based on conventional microfabrication principles and therefore compatible with existing soft lithography and OoC fabrication methods.

### 3.4. Two-Step Fabrication of Compartmentalized Multi-Level Organ-on-a-Chip Devices

**Figure 4:**
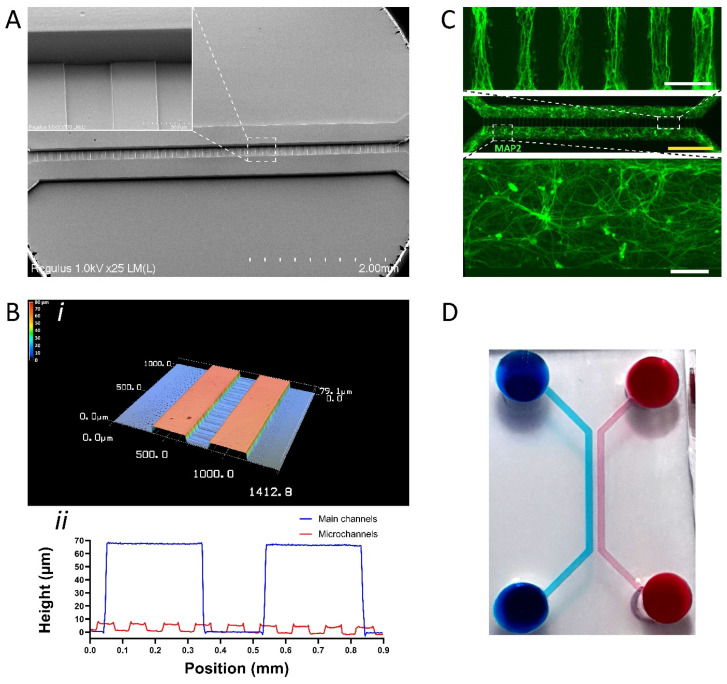
Multi-level SU-8 microstructures to fabricate microfluidic chips with channels of different heights. (**A**) SEM image showing microstructures of a different height. The insert shows an enlarged area of the main channel and perpendicular microchannels of a different height. (**B**) Optical profilometric 3D scan of middle portion of multi-level microfluidic chip. (i) Overview of a 3D scan. (ii) Height measurements of the main and microchannels. (**C**) Representative immunofluorescence image of MAP2-positive hiPSC-derived neurons upon 7 days of culture in a multi-level microfluidic chip. Top dashed box shows an enlarged area of neurite protrusions in microchannels. Bottom dashed box shows an enlarged area of hiPSC-derived neurons cultured in the main channel. (**D**) Photograph of fabricated chip showing fluidic isolation as evident by the blue and red food dyes. White scale bars: 100 µm. Yellow scale bar: 1000 µm.

We have developed a simple method to fabricate structures of different heights that can be used to make compartmentalized chips where two larger cell culture compartments (or channels) are separated by perpendicular microchannels of a different height. This chip design was demonstrated earlier for fluidic isolation of two independent cell-culture compartments and for brain-on-chip studies that require separation of soma from axons, i.e., neurite outgrowth [66,67]. A multi-level SU-8 mold was fabricated using a multi-step SU-8 coating and exposure procedure [56]. By recording the position of the substrate during exposure of the first SU-8 2005 layer with the microchannel patterns, we were able to realign the substrate for the second exposure of a thicker SU-8 2075 layer (described in the Methods section and Supplementary Figure S1). The insert on Figure 4A (dashed line) demonstrates a closer view of SU-8 structures of the main channel (5 mm length, 300 µm width, and 65 µm height) and the microchannels (200 µm length, 50 µm width, and 6 µm height) patterned using a 5X objective.

Optical profilometry of the SU-8 mold (Figure 4B(i)) showed that the microchannel structures were ~6 µm in height (Figure 4B(ii)). Interestingly, we observed that the larger channel structures were ~65 µm in height (Figure 4B(ii)). This is a discrepancy with the thickness of the SU-8 2075 layer, spin coated with a rotational speed of 3000 rpm determined earlier, that should result in a height of 50 µm. This difference might be caused by the reflow effect during the post-exposure bake of the first SU-8 2005 layer resulting in the height differences of the SU-8 2075 layer that was subsequently applied. Importantly, the optical profilometry of the SU-8 mold (Figure 4B(i)) showed that the height was consistent across the entire exposed area despite being higher than expected (i.e., 65 µm vs. expected 50 µm).

To demonstrate the successful fabrication of the multi-level chip, hiPSC-derived neurons were seeded inside the two main channels and cultured for 7 days. Figure 4C shows that the main channels are populated by the neurons after 7 days, as indicated by the neuron-specific MAP2 marker, and that neurites are growing into the lower microchannels. To show fluidic isolation of the main channels, mediated by the lower microchannels, we added red and blue food dyes to each of the main channels of a fabricated chip (Figure 4D).

By using this relatively simple procedure, multi-level SU-8 structures and microfluidic chips with different channel heights can be produced. By using simple realignment and the multi-step SU-8 coating and exposure procedure we described, DMD-based maskless photolithography enables the fabrication of more complex chips for OoC research.

### 3.5. One-Step Fabrication of Multi-Level Microstructures Using Grayscale Photolithography

**Figure 5:**
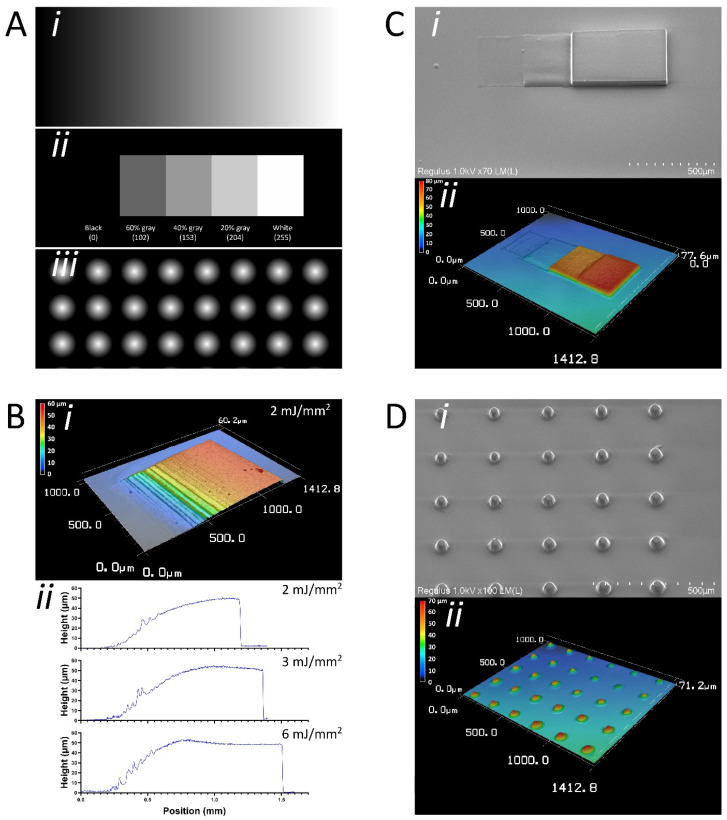
Grayscale photolithography combined with backside UV exposure enables control over microstructure height. (**A**) Digital 8-bit grayscale photomask designs that were used for the grayscale photolithography experiments, using the PRIMO setup and a 5X objective. (i) Linear grayscale gradient to generate SU-8 height gradient. (ii) Staircase-like pattern with various grayscale values to generate multi-level SU-8 structures. (iii) Circles with a radial grayscale gradient to generate cone-like SU-8 structures. (**B**) SU-8 ramp fabricated using linear grayscale gradient as measured by optical profilometry. (i) Structure increases in height from 0 µm to 50 µm. (ii) Near linear gray-height response with a laser dose of 2 mJ/mm^2^. Laser doses that are too high (3 mJ/mm^2^ and 6 mJ/mm^2^) lead to an early plateauing of SU-8 structures. (**C**) Multi-level SU-8 structures can be obtained using grayscale photolithography. (i) SEM image of multi-level SU-8 structure, generated using staircase-like grayscale pattern. (ii) Optical profilometric 3D scan of the multi-level structure. (**D**) Cone-like structures can be fabricated using circles with a radial grayscale gradient. (i) SEM image of cone-like SU-8 structures. (ii) Optical profilometric 3D scan of cone-like SU-8 structures.

Since DMD-based systems can project 8-bit grayscale patterns, we next examined whether multi-level SU-8 structures can be generated in one step by backside UV exposure of SU-8 of 50 µm thickness. Digital photomasks used for SU-8 exposure (using a 5X objective) are shown in Figure 5A. An SU-8 “ramp” that increases in height from 0 µm to ~50 µm was achieved by projecting a grayscale gradient (Figure 5A(i)), as is evident in Figure 5B(i). We determined that a development time of 10 min with a laser dose of 2 mJ/mm^2^ is optimal and provides a near-linear gray-height response (Figure 5B(ii)). The laser dose is crucial because if it is too high (e.g., 6 mJ/mm^2^), there is a loss of control over the height because at that dose the laser provides sufficient energy to fully crosslink SU-8 at lower gray values. This is evident by the early plateauing of SU-8 heights at 3 mJ/mm^2^ and 6 mJ/mm^2^ laser doses (Figure 5B(ii)). In addition, we were able to generate multi-level structures in a single step (Figure 5C(i)). By exposing SU-8 (2 mJ/mm^2^) to a staircase-like pattern consisting of different gray values (Figure 5A(ii)), we obtained a structure that shows a step-wise increase in height at every gray value used (Figure 5C(ii)). Finally, by projecting circles with a radial grayscale pattern (Figure 5A(iii)), we were able to fabricate cone-like structures, as shown in Figure 5D.

In this study, we demonstrated proof-of-principle that the DMD-based PRIMO system can be used for maskless grayscale photolithography using backside UV exposure of SU-8 with PGMEA as a developer agent. Conventional microfabrication methods where photoresists are spin coated onto silicon wafers are not amenable to grayscale photolithography unless conventional exposure and positive photoresists are used [25,27,68]. Maskless grayscale photolithography using SU-8 and glass substrates is a promising strategy for the facile fabrication of complex structures using simplified microfabrication methods [25]. The possibility to control the height of microstructures in a single-step microfabrication process can be beneficial for many OoC applications. For example, it is a potentially faster method to create chips with channels of different heights, as described in Figure 4. Additionally, custom concave microwells could be produced for the generation of microtissues [69], using cone-like or similar structures as demonstrated earlier.

## 4. Conclusions

In this work, we explored the use of low-cost glass coverslips, a negative photoresist, and a commercially available DMD-based setup for maskless UV photolithography (PRIMO, Alvéole Lab). Using established principles, we developed a cleanroom-free microfabrication process flow compatible with soft lithography for rapid prototyping of microfluidic PDMS and OoC devices, with a turnaround time of 6–8 h. We demonstrated that using backside UV exposure, large arrays of microstructures ranging from 20 µm to 100 µm in size, micropillars, and large continuous structures with complex geometries and of different heights could be generated. We further showed that using grayscale photolithography, multi-level structures, and height gradients could be generated in a single-step microfabrication process. Overall, this work demonstrated that SU-8 microstructures can be fabricated with high accuracy on a thin glass substrate using a desktop-sized maskless photolithography system. In addition, chips suitable for cell culture could be quickly produced by using our microfabrication process flow. This rapid prototyping approach is particularly relevant for small volume fabrication of single-material devices as a complement to high-volume batch fabrication of multi-material and multi-functional devices achievable only with cleanroom-based microfabrication techniques.

## Figures and Tables

**Table 1:** Soft bake temperatures and times for SU-8 2075.

Rotational SpeedTemperature	50 °C	65 °C	95 °C
1000 rpm	6 min	15 min	45 min
2000 rpm	4 min	10 min	25 min
3000 rpm	2 min	5 min	12 min
4000 rpm	2 min	5 min	10 min

**Table 2:** Soft bake temperatures and times for SU-8 2005.

Rotational SpeedTemperature	50 °C	65 °C	95 °C
2000 rpm	Not needed	2 min	4 min
4000 rpm	Not needed	2 min	4 min

**Table 3:** Post-exposure bake temperatures and times for SU-8 2075.

Rotational SpeedTemperature	50 °C	65 °C	95 °C
1000 rpm	6 min	12 min	25 min
2000 rpm	4 min	10 min	20 min
3000 rpm	2 min	4 min	10 min
4000 rpm	2 min	4 min	10 min

**Table 4:** Post-exposure bake temperatures and times for SU-8 2005.

Rotational SpeedTemperature	50 °C	65 °C	95 °C
2000 rpm	Not needed	2 min	4 min
4000 rpm	Not needed	2 min	4 min

## Supplementary Materials

The following supporting information can be downloaded at: https://www.mdpi.com/article/10.3390/mi13010049/s1, Figure S1. Substrates can be realigned for fabrication of multi-level chips. (A) The substrate was realigned by using markers that were applied before exposure of the first layer of SU-8. (i) Photograph of an SU-8 coated substrate in the microscope holder. The blue arrows indicate the location of the markers. (ii) Photograph of the applied markers, which span both parts of the substrate and the microscope holder (red arrow indicates part of marker that is on the microscope holder, and blue arrow indicates part of marker that is on the substrate). (B) The exact substrate location was stored by imaging the whole substrate before the first exposure step was performed. (i) Overview image of the scanned substrate, thereby storing the exact location in the Leonardo software. (ii) Landmarks on the substrate (blue arrows) can be used to realign the substrate (using tweezers to carefully move the substrate in the holder) after spin coating of the second layer of SU-8; Figure S2. Flowchart of soft lithography procedure; Figure S3. Digital photomask used for inverted patterns of exposure (Figure 2F); Figure S4. Tall structures ~850 µm in height are possible. (A) Optical profilometric 3D scan of 3:1 aspect ratio pillars with straight sidewalls. (B) SEM image of microstructures with straight sidewalls that can potentially be used to fabricate channels with a larger height for various applications (e.g., organoid trapping); Figure S5. The 2 µm structures can be obtained using the demonstrated process flow. (A) Optical profilometric 3D scan of barcode-like structures of increasing width (2–10 µm), which are 100 µm in length and ~6 µm high. (B) SEM overview image of a small array of barcode-like structures fabricated using a single FoV (20X objective). (C) SEM image of single barcode-like structure. (D) SEM closeup image of 2 µm, 4 µm, and 6 µm wide structures. The 2 µm structures have a 3:1 aspect ratio that increases the tendency of these small structures to deform. Wider structures are not deforming; Table S1. Obtained SU-8 thickness and UV laser doses used at various spinning speeds; Table S2. Duration of the initial step in the process flow (substrate preparation, first row), every subsequent step in the process flow (middle rows), and turnaround time (time until cell seeding, last row). * Applies when rotational speeds of 2000–4000 rpm are used. When rotational speed is <2000 rpm, soft bake time increases significantly. ** Assuming chip design (single-level structures) and substrate preparation (first row) were performed in advance.
