# Supplementary material for: Rapid Prototyping of Organ-on-a-Chip Devices Using Maskless Photolithography
Source: Micromachines (Basel). 2021 Dec 29;13(1):49. doi: 10.3390/mi13010049 (PMC8778126; doi:10.3390/mi13010049)
Supplement: Supplementary file 1 [file micromachines-13-00049-s001.zip › micromachines-1526918-supplementary.pdf]

Supplementary Material: Rapid Prototyping of Organ-on-a-Chip Devices Using Maskless Photolithography

Table S1. Obtained SU-8 thickness and UV laser doses used at various rotational speeds.

Rotational speed	Final thickness	Laser dose
**1000 rpm (SU-8 2075)**	420 µm ± 69 µm	9 mJ/mm^2
**2000 rpm (SU-8 2075)**	81 µm ± 4 µm	7 mJ/mm^2
**3000 rpm (SU-8 2075)**	50 µm ± 2 µm	6 mJ/mm^2
**4000 rpm (SU-8 2075)**	39 µm ± 1 µm	6 mJ/mm^2
**4000 rpm (SU-8 2005)**	~6 µm	17 mJ/mm^2

Table S2. Duration of the initial step in the process flow (substrate preparation, first row), every subsequent step in the process flow (middle rows), and turnaround time (time until cell seeding, last row). *Applies when rotational speeds of 2000–4000 rpm are used. When rotational speed is < 2000 rpm, soft bake time increases significantly. **Assuming chip design (single-level structures) and substrate preparation (first row) were performed in advance.

Step in process flow	Duration
Substrate preparation (spin coating and soft bake)(performed in bulk)*	60 min (for 8 substrates)
Exposure	10 min
Post-exposure bake	30–50 min
Development	10 min
Soft lithography (mounting of substrate, PDMS curing, and chip assembly)	200 min
Coating of chip with proteins of interest	120–180 min
Turnaround time (time until cell seeding)**	**6–8 h**

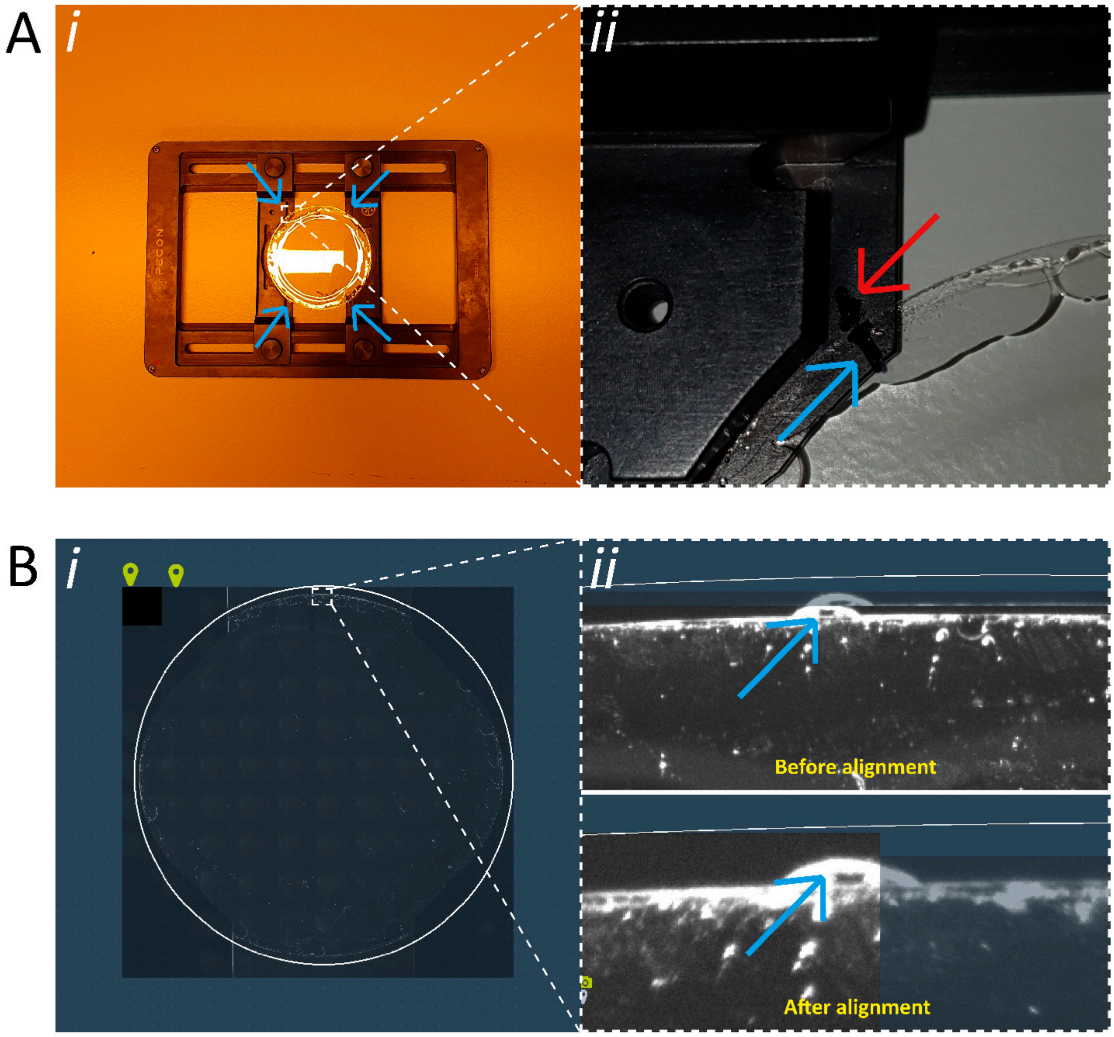

Figure S1. Substrates can be realigned for fabrication of multi-level chips. (A) The substrate was realigned by using markers that were applied before exposure of the first layer of SU-8. (i) Photograph of an SU-8 coated substrate in the microscope holder. The blue arrows indicate the location of the markers. (ii) Photograph of the applied markers, which span both parts of the substrate and the microscope holder (red arrow indicates part of marker that is on the microscope holder, and blue arrow indicates part of marker that is on the substrate). (B) The exact substrate location was stored by imaging the whole substrate before the first exposure step was performed. (i) Overview image of the scanned substrate, thereby storing the exact location in the Leonardo software. (ii) Landmarks on the substrate (blue arrows) can be used to realign the substrate (using tweezers to carefully move the substrate in the holder) after spin coating of the second layer of SU-8.

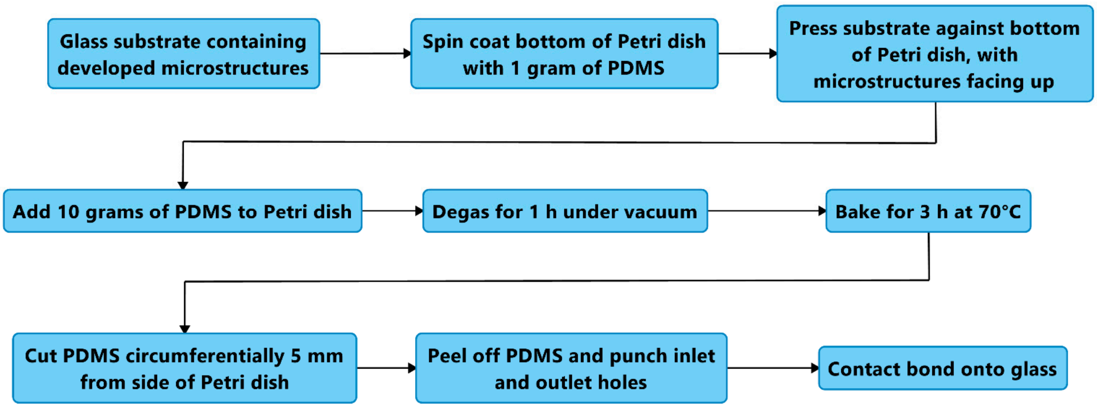

Figure S2. Flowchart of soft lithography procedure.

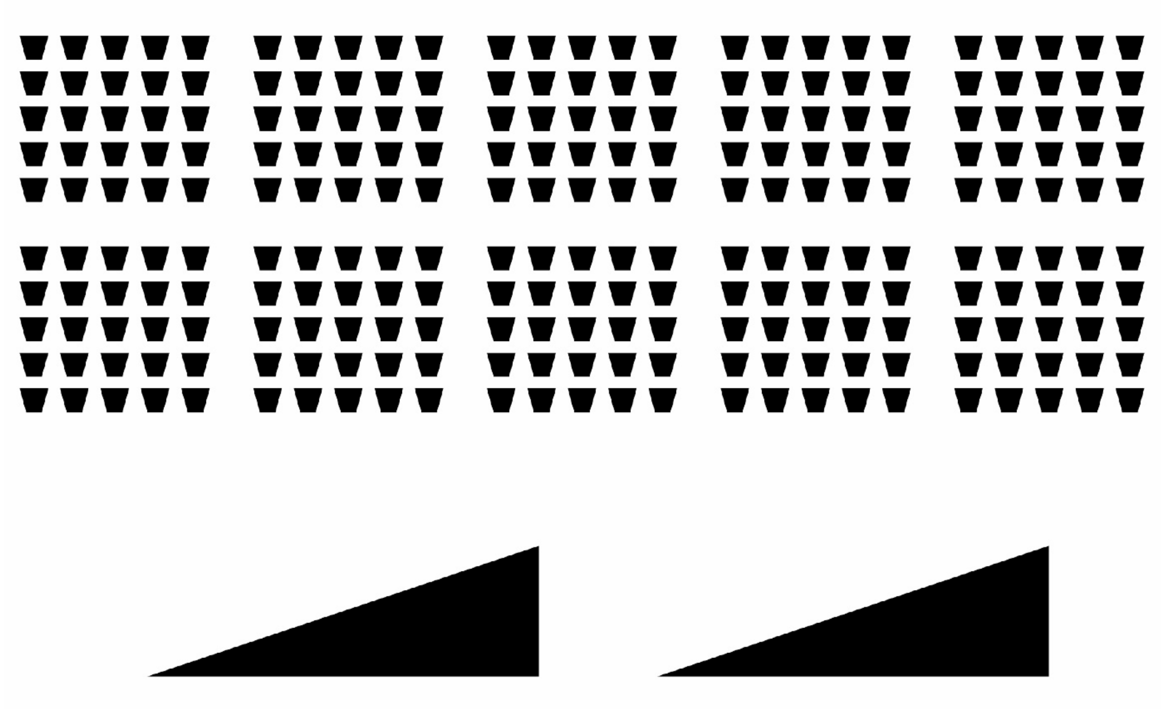

Figure S3. Digital photomask used for inverted patterns of exposure (Figure 2F).

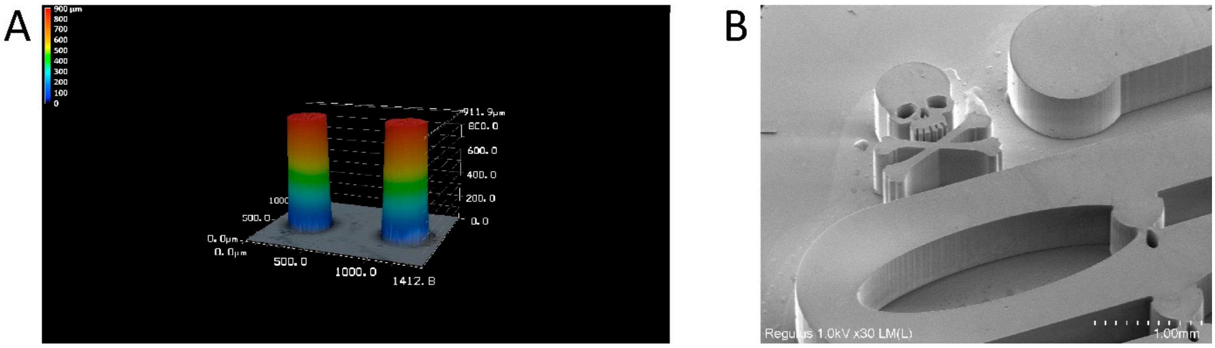

Figure S4. Tall structures ~850 µm in height are possible. (A) Optical profilometric 3D scan of 3:1 aspect ratio pillars with straight sidewalls. (B) SEM image of microstructures with straight sidewalls that can potentially be used to fabricate channels with a larger height for various applications (e.g., organoid trapping).

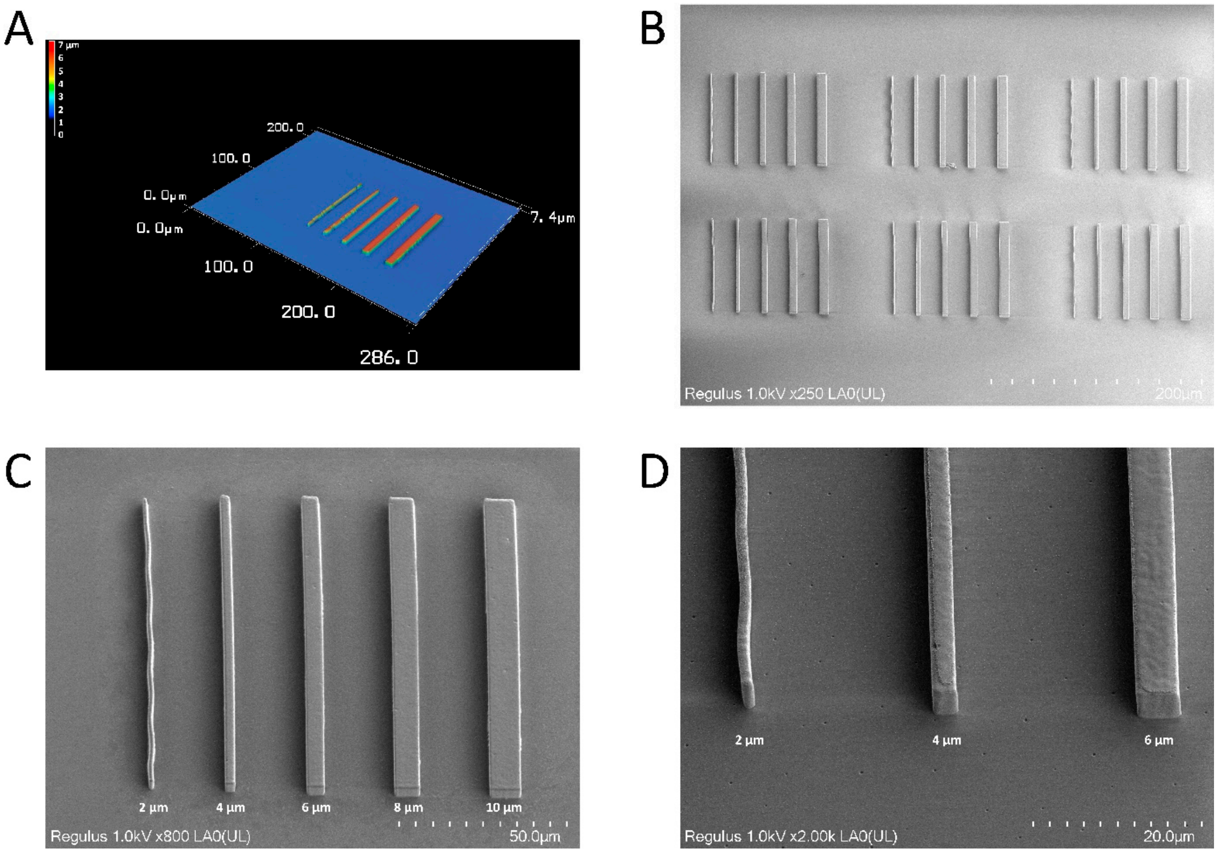

Figure S5. The 2 µm structures can be obtained using the demonstrated process flow. (A) Optical profilometric 3D scan of barcode-like structures of increasing width (2–10 µm), which are 100 µm in length and ~6 µm high. (B) SEM overview image of a small array of barcode-like structures fabricated using a single FoV (20X objective). (C) SEM image of single barcode-like structure. (D) SEM closeup image of 2 µm, 4 µm, and 6 µm wide structures. The 2 µm structures have a 3:1 aspect ratio that increases the tendency of these small structures to deform. Wider structures are not deforming.
